# Treatment of tobacco addiction using the Feeling-State Addiction Protocol (FSAP) of the Eye Movement Desensitization and Reprocessing (EMDR) treatment

**DOI:** 10.1186/1617-9625-12-S1-A25

**Published:** 2014-06-06

**Authors:** Aikaterini Tsoutsa, Dimos Fotopoulos, Spyridon Zakynthinos, Paraskevi Katsaounou

**Affiliations:** 1 Pulmonary &Critical Care Department, Evaggelismos Hospital, Athens, 10676, Greece

## Background

Compulsions and cravings for smoking have been the subject of behavioral treatment. EMDR [1] is an established, effective treatment of trauma-based disorders [2]. Its use in the treatment of addictions and compulsions is relatively new. Although there are ways of targeting irrational positive affect via EMDR [3]. Merging the Feeling-State Theory of Compulsions and EMDR, the Eye Movement Compulsion Protocol (EMCP) was developed. EMCP is used for fading both feelings and un-wanted behavior related to smoking. The FSAP hypothesizes that the pleasure during smoking is imprinted in the brain generating feelings like comfort, contentment and happiness [4].Thus, when craving resurges, the Feeling-State (FS) behavior is re-enacted. The EMCP incorporates the standard eye movement technique of EMDR to reduce the FS associated with impulsion to smoke. This study aims to assess the efficacy of the FSAP in the treatment of tobacco addiction of relapsed smokers with persistent compulsions to smoke

## Materials and methods

We studied 2 groups (12 smokers in each), that relapsed (at least 1 m after smoking cessation). Smokers were matched for age, sex, Fagerstrom Test for Nicotine Dependence & pack/d.

## Results

The FSAP although brief, results in profound changes in behavior [4]. Consequently, the 1st group was administered 6 sessions of the FSAP protocol. The 2nd group had 6 sessions of Cognitive Behavior Therapy. The 2 groups were compared for smoking cessation (self-reported questionnaire, CO-measurements). The 1st group had a succession rate of 50% vs the second that had only 25%.

## Conclusion

Thus, we conclude that EMDR could be a very helpful tool in managing smoking relapses.

## References

[B1] Shapiro F Eye movement desensitization and reprocessing (EMDR): Basic principles, protocols, and procedures 2001 2 New York: Guilford Press

[B2] Van der Kolk B Spinazzolla J Blaustein M Hopper E Korn D Simpson W A randomized clinical trial of EMDR, fluoxetine and pill placebo in the treatment of PTSD: Treatment effects and long-term maintenance Journal of Clinical Psychiatry 2007 68 37 46 10.4088/JCP.v68n0105 17284128

[B3] Knipe J R. Shapiro Targeting positive affect to clear the pain of unrequited love, codependence, avoidance, and procrastination EMDR solutions: Pathways to healing 2005 New York: W W Norton & Co 189 212

[B4] Miller R The feeling-state theory of impulse-control disorders and the impulse-control disorder protocol Traumatology 2010 16 3 2 10

